# Epidemiological insights into canine rabies in Chennai: Trends, forecasting and One Health implications^☆^

**DOI:** 10.1016/j.onehlt.2025.101128

**Published:** 2025-07-04

**Authors:** Viswanathan Naveenkumar, Mangalanathan Vijaya Bharathi, Porteen Kannan, B.S. Pradeep Nag, Sureshkannan Sundaram, Nithya Quintoil Mohanadasse, Raghavendra G. Amachawadi, Muskan Dubey, Charley A. Cull, Chandan Shivamallu, Shiva Prasad Kollur, Ravindra P. Veeranna

**Affiliations:** a Veterinary Clinical Complex, Veterinary College and Research Institute, Tamil Nadu Veterinary and Animal Sciences University (TANUVAS), Udumalpet, Tiruppur 642 205, Tamil Nadu, India; b Department of Veterinary Public Health and Epidemiology, Veterinary College and Research Institute, Tamil Nadu Veterinary and Animal Sciences University (TANUVAS), Salem 636 112, Tamil Nadu, India; c Department of Veterinary Public Health and Epidemiology, Madras Veterinary College, Tamil Nadu Veterinary and Animal Sciences University (TANUVAS), Chennai 600 007, Tamil Nadu, India; d Department of Animal Sciences, University of Missouri, Columbia 65211, MO, United States; e Department of Veterinary Public Health and Epidemiology, RIVER, Pondicherry 605 009, India; f Department of Clinical Sciences, College of Veterinary Medicine, Kansas State University, Manhattan, KS 665065606, USA; g Xavier University School of Medicine, Santa Helenastraat #23, Oranjestad, Aruba; h Midwest Veterinary Services, Inc., a part of the Argenta Group, Oakland, NE 68045, USA; i Department of Biotechnology and Bioinformatics, JSS Academy of Higher Education and Research, Mysuru 570 015, Karnataka, India; j School of Physical Sciences, Amrita Vishwa Vidyapeetham, Mysuru Campus, Mysuru 570 026, Karnataka, India; k Xavier University School of Veterinary Medicine, Santa Helenastraat #23, Oranjestad, Aruba

**Keywords:** Canine rabies, Epidemiology, Longitudinal studies, Trend analysis, Change point analysis, Time series prediction

## Abstract

Eliminating canine-mediated human rabies deaths by 2030 is a global priority, necessitating a data-driven approach to understand rabies dynamics and implement effective prevention strategies. This study provides epidemiological insights into canine rabies in Chennai, analyzing nine years of surveillance data (*n* = 428, March 2010 – February 2019) to assess trends, seasonality and predictive patterns. Change point and time series analyses were conducted and forecasting models were evaluated using Root Mean Squared Error (RMSE), Mean Absolute Error (MAE), Mean Absolute Percentage Error (MAPE) and Mean Absolute Scaled Error (MASE) metrics. No significant seasonality was detected, but change point analysis identified two key shifts, segmenting the data into three phases and revealing an overall declining trend. Among the models tested, the Prophet model demonstrated the best predictive performance (RMSE: 1.88, MAE: 1.55, MAPE: 45.44 %, MASE: 3.52), outperforming the Generalized Additive Model (GAM), Bayesian Structural Time Series (BSTS) and Seasonal Trend decomposition using Loess combined with ARIMA (STL + ARIMA (0,0,2)). This study offers critical epidemiological insights for strengthening One Health-based rabies control strategies, particularly in urban settings where canine rabies plays a major role in human exposure risk. By providing longitudinal data and predictive modelling, these findings guide targeted preventive interventions, inform evidence-based policy decisions and support global efforts to eliminate dog-mediated human rabies deaths by 2030.

## Graphical abstract

**Figure:**
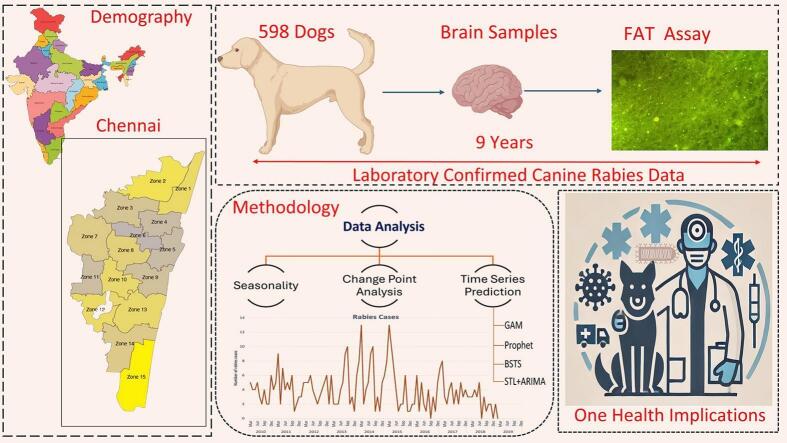

## 1 Introduction

Rabies is among the deadliest viral zoonotic diseases, impacting both animals and humans with a 100 % case fatality rate. It poses a significant public health concern across over 150 countries and territories, particularly in Asia and Africa [1]. Despite over 4300 years of rabies history, only 29 human cases of recovery from infection have been recorded globally [2]. Annually, approximately 59,000 people succumb to rabies worldwide, with over 36 % of these deaths occurring in India. Globally, rabies transmission is classified into two primary types: urban (canine-mediated) and sylvatic (wild animal-mediated). In developing countries such as India, dogs serve as the principal reservoir, accounting for 99 % of human rabies cases due to dog-mediated transmission [3]. Effective vaccination in dogs is therefore a crucial component of control strategies for both animal and human rabies. Despite the severity of rabies, the implementation of regulatory standards for rabies control requires greater attention, contributing to the disease's neglected status and poor documentation.

In India, the widespread practice of community-based or free-ranging dog rearing poses unique challenges in devising and implementing effective rabies prevention strategies. Limited access to standard laboratories for rabies diagnosis increases the risk of rabies transmission from suspected cases worldwide [[4], [5], [6]]. Community based awareness is considered a top priority in achieving the goal of “elimination of dog mediated human rabies deaths by 2030”, a noble cause set by global health agencies. Documenting current trends and region-specific surveillance data is essential for this effort, providing baseline data for policymakers, government and non governmental organizations. Understanding rabies occurrence patterns in canines is essential for establishing preventive strategies and identified trends can serve as key elements in evaluating and adjusting existing strategies [3]. While cross sectional studies on animal rabies cases have been conducted globally, there is a scarcity of long-term studies with laboratory confirmed rabies data. We hypothesize that long-term surveillance data will uncover significant trends and shifts in rabies incidence that reflect the implementation status of control measures, thereby helping to evaluate the sustained impact of existing strategies and guide the refinement of future interventions. In this study, a longitudinal approach was used with nine years of laboratory confirmed canine rabies data from southern India.

In data analysis, predicting future events based on historical data is widely applicable across fields, including medical and veterinary sectors, where prediction models are increasingly vital for understanding infectious disease epidemiology [3]. Time series models are among the most well established and commonly used methods for identifying patterns in infectious disease data. These models can identify temporal dependencies within datasets, capturing trends, seasonality and other patterns [7]. Various time series models have been documented in data science. We further hypothesize that advanced time series models can accurately capture and forecast fluctuations in rabies cases and that such models will identify meaningful change points that align with shifts in disease dynamics. These insights could indirectly reflect the impact of field-level interventions or emerging epidemiological risks. In this study, like these four models were used to predict rabies cases based on past laboratory confirmed data: (i) Generalized Additive Models (GAM) provide flexible, non linear trend modelling for complex patterns; (ii) Prophet automatically detects seasonality and events such as holidays; (iii) Bayesian Structural Time Series (BSTS) captures structural shifts and uncertainties through Bayesian inference; and (iv) Seasonal Trend decomposition using Loess combined with the Autoregressive Integrated Moving Average (STL + ARIMA) effectively combines seasonal decomposition with robust forecasting. Each model has unique strengths and limitations for predicting future events based on temporal dependencies in past observations. Additionally, change point analysis was used to detect abrupt shifts in the time series data, aiding in trend interpretation.

With this background, the present study aimed to (i) identify trends and seasonality in canine rabies epidemiology in Chennai, India, using nine years of laboratory confirmed data (March 2010 to February 2019) through change point and time series analyses and (ii) compare the performance of different time series models (GAM, Prophet, BSTS and STL + ARIMA) in predicting canine rabies cases.

## 2 Materials and methods

### 2.1 Data source

The study was conducted in Chennai, the capital of Tamil Nadu, situated on the southeastern coast of India along the Bay of Bengal, at approximately 13.08°N latitude and 80.27°E longitude. A total of 428 laboratory confirmed canine rabies cases over nine years (March 2010 to February 2019) was used in this study. Laboratory confirmation includes fluorescent antibody test (FAT) and/or Sellers staining of suspected dogs brain samples. The data was collected and analyzed from the Teaching Veterinary Hospital, Madras Veterinary College, Chennai, India. Detailed data collection and sampling inclusion criteria were provided in our earlier publications [3].

### 2.2 Study period and descriptive analysis

The occurrences of canine rabies data collected for the entire period (March 2010 to February 2019) were tabulated in Microsoft Excel 2016 (monthly data). Further, all statistical analyses were carried out in R software (version 4.3.1).

### 2.3 Change point analysis

To identify key changes in rabies occurrences from March 2010 to February 2019, change point analysis was applied to the time series data. Given that the data represent counts of rabies cases, a Poisson distribution was assumed, which is commonly used for count-based datasets in epidemiological studies [[8], [9], [10]]. To further justify this choice, we assessed the data for overdispersion by comparing the mean and variance of monthly rabies case counts. Although mild overdispersion was observed, it remained within acceptable limits for this application. Since the primary objective of this analysis was to detect structural changes in the time series rather than estimate parameters with precision, the Poisson distribution was considered appropriate [9]. Moreover, the binary segmentation was implemented using the ‘*cpt.meanvar*’ function from the “changepoint” package in R, which supports Poisson-distributed input data and is robust for detecting changes in both mean and variance. We utilized a binary segmentation technique to detect change points within the data, allowing the time series to be split into segments with distinct statistical properties [11]. The mathematical equation used in this study for the detection of change points is detailed in the Supplementary material. The identified change points provided insights into shifts in the trends of rabies occurrences, allowing us to investigate potential factors contributing to these changes over the study period.

### 2.4 Seasonality test

To examine seasonality in the time series dataset, we used the Ollech-Webel overall seasonality test (WO test) from the “*seastests*” package. The WO test integrates results from both the QS test and the Kruskal-Wallis (KW) test. The QS test detects seasonality by analyzing autocorrelation in seasonal lags, while the KW test uses a non parametric, rank based method to assess monthly data distributions [10,12]. Seasonality is indicated by the WO test if the QS test *p*-value is below 0.01 or if the KW test p-value is below 0.002. For this study, we categorized seasons based on local climate patterns: Winter (January and February), Summer (March to May), Southeast Monsoon (June to September) and Northeast Monsoon (October to December).

### 2.5 Time series analysis

For the development of the time series model, the following steps were performed: (i) decomposition of time series data into trend and seasonality; (ii) development of different prediction models (GAM, Prophet, BSTS and STL + ARIMA); and (iii) comparison of the predictive performance of developed models using model construction and validation datasets. From the available data (March 2010 to February 2019), the dataset was divided into training and validation sets. The training dataset includes data from March 2010 to February 2018 (96 monthly observations), while the validation dataset includes data from March 2018 to February 2019 (12 months).

### 2.6 Decomposition of time series data

To analyze the trend and seasonal patterns of animal rabies occurrences, an additive decomposition model was applied. This approach separates the time series into three key components: trend, seasonality and remainder. In this study, decomposition was performed using a 12-month cycle and the model employed for this purpose is described in the Supplementary material. This decomposition enables us to assess both the overall trend and seasonal variations in rabies case occurrences across the study period.

### 2.7 Development of time series models

#### 2.7.1 Generalized Additive Model (GAM)

The Generalized Additive Model (GAM) is a flexible approach to regression that accommodates both linear and non-linear relationships, making it suitable for complex datasets like those involving disease trends. In this study, GAM was used to analyze trends in rabies cases over time, capturing non-linear patterns through smooth functions and reducing assumptions on the relationship between predictor variables and response, thereby achieving high predictive accuracy. Using “*mgcv”* package in R, the GAM model was built following previously published methods [13,14]. The structure of the GAM and the exponential family distribution used in the model are described in the Supplementary material.

#### 2.7.2 Prophet model

The Prophet model, an open-source forecasting tool developed by Facebook in 2018 [15], was used to analyze and forecast monthly rabies cases. Prophet is designed for univariate time series data, automatically modelling key components such as trend, seasonality and noise. The analysis was conducted using the “*prophet”* package in R statistical language. The equations underlying the Prophet model are detailed in the Supplementary material.

#### 2.7.3 Bayesian Structural Time Series (BSTS)

The Bayesian Structural Time Series (BSTS) model was employed to forecast monthly rabies cases. Unlike traditional time series methods, such as ARIMA, which rely solely on past values, BSTS allows model parameters to vary over time, providing greater flexibility in capturing complex, evolving epidemic patterns. BSTS also incorporates multiple components such as trend and seasonality while managing uncertainty through Bayesian inference, making it particularly suitable for disease incidence data [16,17]. The model incorporated a trend component and seasonality, capturing long term patterns and periodic fluctuations in rabies incidence. The equations pertaining to the BSTS model construction are provided in the Supplementary material. The BSTS model was implemented using the “*bsts”* package in R, with the trend and seasonality components included. The model was run with 1000 iterations to provide posterior estimates of the trend and seasonality, capturing the underlying structure of the rabies case data. The Kalman filter was used to estimate trends and seasonal behaviour and Bayesian inference quantified the uncertainty in these components.

#### 2.7.4 Seasonal and Trend Decomposition using Loess (STL) combined with an ARIMA model (STL + ARIMA)

To model and forecast monthly rabies cases, we applied the Seasonal and Trend Decomposition using Loess (STL) combined with an ARIMA model, referred to as the STL + ARIMA approach [18]. STL is a robust, non parametric decomposition method that uses locally weighted scatterplot smoothing (loess) to iteratively estimate and refine the trend and seasonal components, handling potential outliers effectively. In this study, STL was first applied to decompose the rabies case time series into trend, seasonal and remainder components. After decomposition, an ARIMA model was fitted specifically to the remainder component *R*_*T*_, capturing any residual autocorrelation that the trend and seasonality did not explain.

The STL + ARIMA approach offers several advantages. By removing seasonality and trend before fitting ARIMA, it reduces the risk of overfitting and improves forecast accuracy, especially for data with strong seasonal patterns. Following decomposition, the ARIMA model forecasts the residual component, which is then combined with the seasonal and trend components to produce the final forecast [19]. The mathematical equations pertaining to the STL + ARIMA approach are provided in the Supplementary material.

The ‘*auto.arima’* function, part of the “*forecast”* package, automatically selected the optimal ARIMA order for the residual component based on Akaike Information Criterion (AIC). The final forecast was derived by adding the predicted trend, seasonal and residual components.

### 2.8 Forecast and model performances

The developed models were evaluated based on their ability to predict unknown data, with comparison to the test data (validation dataset) from March 2018 to February 2019. The evaluation metrics used to compare the prediction models included root mean squared error (RMSE), mean absolute error (MAE), mean absolute percentage error (MAPE) and mean absolute scaled error (MASE) [19,20]. Among the various models, the one with the lowest error values was considered the best fit for the dataset [21]. Further, forecasting was performed for the developed model for the period from March 2019 to February 2020. Data visualization and forecasting were conducted in R using the “*ggplot2*” and “*forecast*” packages, respectively.

## 3 Results

### 3.1 Canine rabies descriptive analysis

From the nine-year dataset (March 2010 to February 2019), a total of 428 laboratory confirmed cases out of 598 suspected dog brain samples were included, yielding a positivity rate of 71.57 % for this time series study. Descriptive analysis revealed that across the 108 months of observation, the average case count was 3.96 per month (SD = 2.63), with a maximum of 13 cases and a minimum of 0 cases in a single month (Table 1). The interquartile range indicated that 50 % of cases fell between 3 and 5 per month, highlighting moderate variability in rabies incidence over the months.

**Table 1:** Descriptive statistics of the monthly incidence of canine rabies in Chennai from March 2010 to February 2019.

Variable	Number of months	Mean	SD	Min	P25	P50	P75	Max	IQR⁎
Canine Rabies	108	3.96	2.63	0.00	2.00	4.00	5.00	13.00	3.00

⁎ IQR: interquartile range P75 – P25.

### 3.2 Change point analysis

**Fig. 1:**
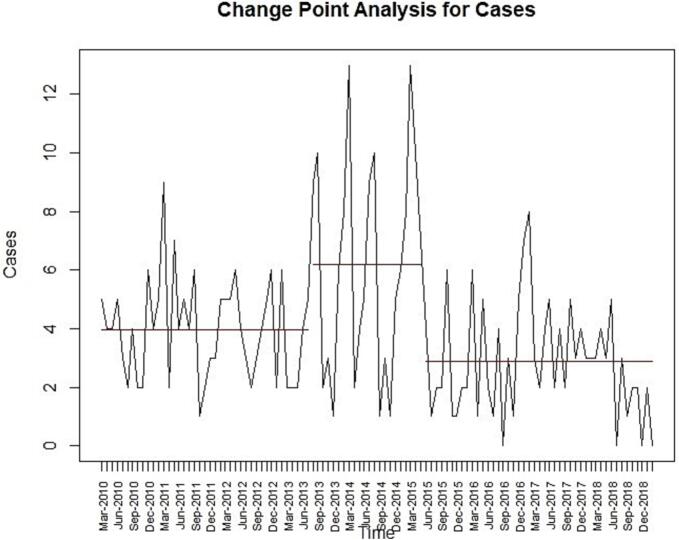
Change point analysis of canine rabies incidence in Chennai: time series data from March 2010 to February 2019.

Change point analysis of the canine rabies time series data identified two significant change points (July 2013 and May 2015), resulting in three distinct segments of rabies incidence trends in Chennai (Fig. 1). After a stable initial segment, the second segment was shorter (one year and ten months) and marked by peak caseloads in March 2014 and March 2015. Following the second change point, the third segment showed a decreasing trend with cases stabilizing at lower levels.

### 3.3 Seasonality test

Seasonality testing, with *p* values of 0.0926 (QS test), 0.1683 (QS R test) and 0.0668 (KW R test), indicated no statistically significant seasonal pattern within the canine rabies time series data.

### 3.4 Trend analysis

**Fig. 2:**
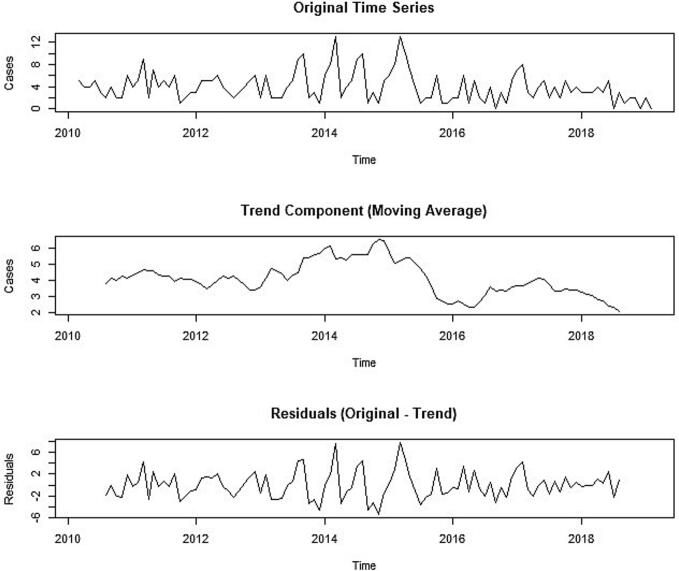
Additive decomposition of the canine rabies time series data using non seasonal trend residual approach.

Additive decomposition of the data using a non seasonal trend residual approach (Fig. 2) highlighted distinct phases in rabies case trends over the study period. The initial phase, from March 2010 to July 2013, showed a stable caseload, followed by an increasing trend peaking in May 2014 and May 2015, with the highest recorded monthly case count (*n* = 13), marking an epidemic peak within the dataset. After mid of 2015, case numbers declined and remained consistently low with minimal fluctuations. No cyclical patterns in rabies cases were observed across specific months or seasons. The random component, which represents residual variations after trend removal, showed fluctuations, with larger residuals during the peak period (2013–2016) that tapered as trends stabilized in later years.

### 3.5 Prediction models and performance comparison

**Fig. 3:**
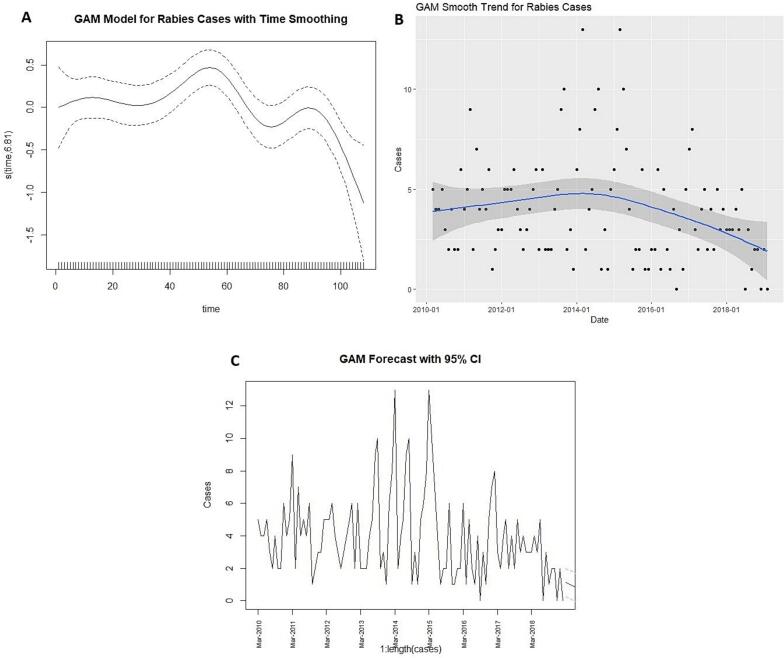
GAM model results. (A) Developed GAM model layout results with time smoothing plot. (B). Developed GAM model, with a time smoothing trend plot. (C). GAM model forecast plot with 95 % confidence interval.

Univariate time series models (GAM, Prophet, BSTS and STL + ARIMA) were developed to forecast future trends in canine rabies in Chennai, Southern India. The GAM model, with a time smoothing plot depicted in Fig. 3, revealed fluctuations over time, with an initial increase and peak during 2014–2015, followed by a downward trend toward the series end. The forecast plot suggests a continued decline in rabies cases, with narrow confidence intervals indicating stable and reliable predictions (Fig. 3).

**Fig. 4:**
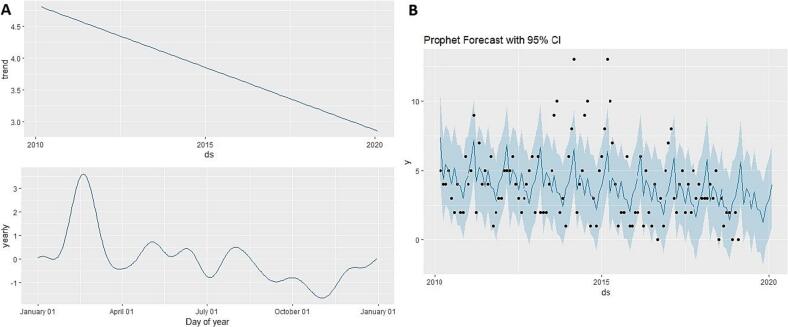
Prophet model results. (A). The component plot of Prophet model. (B). Prophet model forecast plot with 95 % confidence interval.

**Fig. 5:**
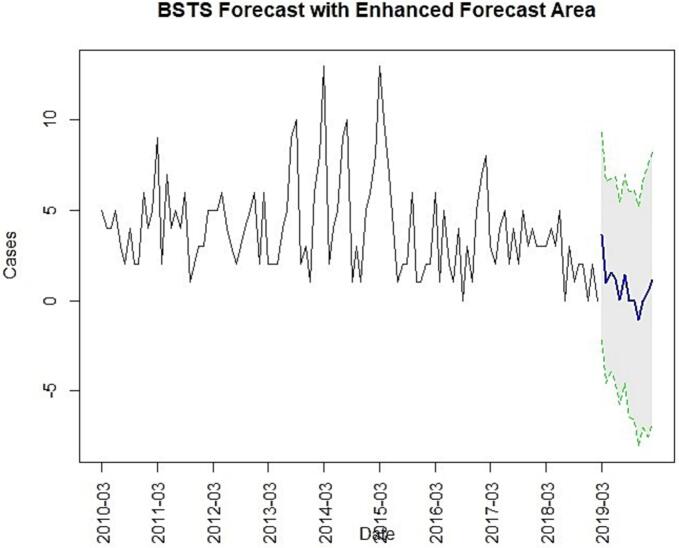
BSTS model forecast with 95 % confidence interval.

The Prophet model analysis showed a downward trend from 2010 to 2020, indicating a gradual decline in rabies cases over the study period. The component plot suggests a yearly seasonal trend with a peak around the early months of the year (February and March) and some fluctuations throughout the rest of the year, despite the earlier seasonality test showing no statistically significant seasonality (Fig. 4). The forecast plot fit well with the observed data, though it displayed a wide 95 % confidence interval during the mid study period (2014 to 2015), reflecting uncertainty during peak periods (Fig. 4). The BSTS forecast plot (Fig. 5) displayed a wide 95 % confidence interval in the forecast area, increasing uncertainty with stable low cases in the mid forecast.

**Fig. 6:**
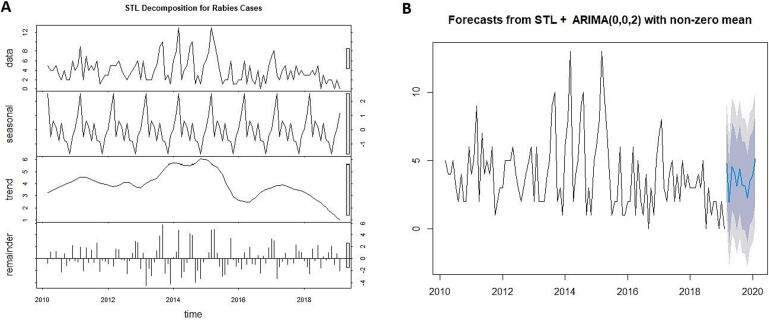
STL + ARIMA (0,0,2) model results. (A). STL decomposition plot with remainder, seasonal and trend component. (B). STL + ARIMA (0,0,2) Model forecast plot with 95 % confidence interval.

The STL + ARIMA (0,0,2) model forecast plot (Fig. 6) suggested minor fluctuations within the forecast range. STL decomposition breaks down the data into trend, seasonal and remainder components (Fig. 6). The trend component reveals a gradual decline after the peak in 2014–2015, while the seasonal plot suggests recurring patterns with peaks, particularly early in each year (February and March months). The remainder component captures random variations, with notable spikes during the increased trend years (2014 and 2015).

**Fig. 7:**
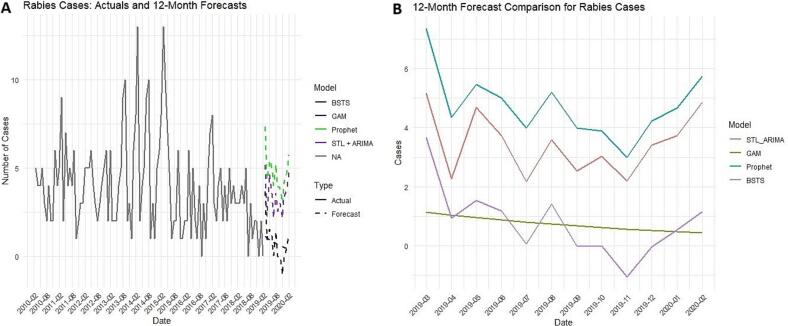
Forecast comparison of various prediction models. (A). Actual and forecast comparison of various prediction models. (B). 12-Month forecast comparison of various prediction models.

Comparison of the models was performed, with evaluation metrics presented in Table 2. Prophet was found to be the optimal model for this dataset with error metrics (MAE – 1.55, RMSE – 1.88, MAPE – 45.44 % and MASE – 3.52), followed by GAM, BSTS and STL + ARIMA (0,0,2). The forecast comparison plot showed distinct trends across the models (Fig. 7). The STL + ARIMA and Prophet models suggested a slight increase in cases toward the early months of 2020, whereas GAM indicated stability and BSTS projected a gradual decline.

**Table 2:** Predictive performance comparison of models for canine rabies prediction.

Model	Mean absolute error (MAE)	Root mean squared error (RMSE)	Mean absolute percentage error (MAPE %)	Mean absolute scaled error (MASE)
Generalized Additive Model (GAM)	1.64	1.96	68.07	3.85
Prophet	1.55	1.88	45.44	3.52
Bayesian Structural Time Series (BSTS)	1.61	1.97	77.70	3.86
Seasonal and Trend decomposition using Loess + Autoregressive Integrated Moving Average model (STL + ARIMA (0,0,2))	2.07	2.42	67.64	5.86

## 4 Discussion

Understanding the pattern of canine rabies through laboratory confirmed cases is crucial for estimating trends, seasonality and other disease occurrence patterns [3]. Globally, long term studies using advanced time series analysis for rabies are limited. In countries like India, the role of dogs in the rabies transmission chain is well documented; however, long term laboratory confirmed data on canine rabies are scarce [22]. During this study period, nine years of data from clinically suspected dogs (*n* = 598), with a laboratory confirmed incidence rate of 71.57 %, suggested an endemic pattern of canine rabies in Chennai, Tamil Nadu, India. The finding of rabies endemicity in Chennai (85.1 %) was supported by an earlier study conducted between 2011 and 2014 [23]. This study further explores long term confirmed cases to identify trends and seasonality through change point analysis and time series modelling using GAM, Prophet, BSTS and STL + ARIMA techniques. Our earlier study used a basic ARIMA model with exogenous meteorological variables to explore rabies trends in Chennai [3]. However, to address non-linear patterns, structural shifts and uncertainty in long-term surveillance data, this study employed more advanced models such as GAM, Prophet, BSTS and STL + ARIMA. These models provided a more comprehensive understanding of rabies seasonality, trend shifts and forecasting performance beyond the capabilities of conventional ARIMA models. Advanced time series analysis (using GAM, Prophet and BSTS) combined with a hybrid approach (STL + ARIMA) revealed patterns, trends, randomness and significant insights into the dataset. Descriptive analysis, with interquartile range results showing that 50 % of cases fall between 3 and 5 per month, indicates substantial monthly variation, supporting the use of this data in time series analysis.

Our previous study published a descriptive statistical understanding of canine rabies for the study period [3]. This present study dives deeper, focusing on trends, seasonality and occurrence patterns using time series data. Change point analysis aids in identifying trends and segments data with similar variance and mean. In this study, two significant change points were identified, resulting in three distinct data segments. In a similar fashion, a longitudinal study in Nepal analyzed rabies time series data from 2005 to 2018 and identified three change points with four data segments [10]. Although no strong seasonal pattern was identified in the present study, this outcome may be influenced by the limited sample size in some months, potential reporting bias, or the inherent sensitivity of the models used, which may not capture weak or irregular seasonal effects in datasets with moderate overdispersion [12]. The non-seasonal pattern of rabies occurrence observed here aligns with the earlier study [10], which analyzed rabies occurrence patterns for both livestock and canine data, whereas our findings are specific to the canine pattern in Chennai, Tamil Nadu. Canine mediated rabies is the predominant transmission vector in Asia, accounting for 95–99 % of cases; both studies reflect similar results in seasonality analysis, though factors like geography, host availability and preventive strategies also contribute.

Similar trends have been documented in other urban settings globally, reinforcing the generalizability of our findings. For instance, a 19-year retrospective analysis in Nigeria (2000–2018) reported a 64 % incidence of canine rabies in urban and semi-urban regions, with fluctuating trends influenced by dog population dynamics, diagnostic capabilities and variations in control strategies [24]. Likewise, a comprehensive literature review and meta-analysis on rabies surveillance across Asia noted that between 2010 and 2024, dogs accounted for a test prevalence of 38.1 %, second only to foxes, highlighting their predominant role in disease transmission in the region [25]. In this context, the 71.57 % laboratory-confirmed incidence observed in our nine-year Chennai dataset underscores a substantial disease burden. Together, these findings from Nigeria, broader Asia and the current study illustrate consistent canine rabies patterns across diverse urban environments, emphasizing the need for sustained surveillance and integrated control efforts globally.

Trend analysis revealed a decline in cases over the nine-year observation period. Limited data on animal cases (suspected, probable, confirmed and absent cases) pose challenges for comparative analysis, potentially leading to an underestimation of disease burden and gaps in strategy implementation [4,5]. Publicly available human rabies data could provide a valuable comparison platform. For instance, a national secular trend analysis of human rabies data in India from 2005 to 2020 revealed a decline in incidence, from 2.36 to 0.41 per 10 million population [26]. In this human rabies study, choropleth mapping showed Tamil Nadu's incidence at 0.1 to 1, while other Indian states reached up to 6.5. This declining pattern in both Indian and Tamil Nadu human rabies data aligns with our canine rabies data.

The historical developments influencing canine rabies trends in Tamil Nadu, particularly in Chennai, were compared with segmented data differences from this study. In Tamil Nadu, Blue Cross India launched the Animal Birth Control Programme combined with Anti Rabies (ABC-AR) in 1964, with systematic implementation beginning in 1996. Tamil Nadu pioneered state wide multisectoral rabies control initiatives, integrating human, animal and other sectors under One Health through structured committees and frameworks by the end of the first decade of the 21st century [27]. These efforts began in 2011 and our study's first data segment (March 2010 to July 2013) revealed a stable case load in Chennai. In the second segment, increased uncertainty and two peaks (March 2014 and March 2015) highlight the need for exploratory studies on vaccine loads, dog bite cases, confirmed cases and other factors. A case study conducted in Tamil Nadu [28] reported 65 human rabies fatalities in the state during the year 2014. According to National Centre for Disease Control (NCDC), Government of India data, from 2012 to 2022, Tamil Nadu recorded 155 human rabies deaths [29]. In 2014 alone, 65 cases were documented, indicating a cluster in Tamil Nadu's rabies data consistent with our findings of increased uncertainty.

During the study period, the final trend showed a decline from mid of 2015 to the end of the study (February 2019). In India, the National Rabies Control Programme (NRCP) was implemented in 2013, with a pilot study in the animal component conducted in Chennai and Haryana from 2014 to 2017 (https://ncdc.mohfw.gov.in/national-rabies-control-programme/). Tamil Nadu's commitment to the National Action Plan for Rabies Elimination by 2030 (NAPRE) led to mandated 24/7 post exposure prophylaxis and a minimum stock of 20 anti rabies vaccine vials in government hospitals including primary health centre [30,31]. These coordinated efforts since 2014 may contribute to the declining trend in canine rabies in Chennai. Although the observed decline in rabies incidence after 2015 aligns temporally with the implementation of control initiatives such as the NRCP and NAPRE in Tamil Nadu, causal relationships cannot be confirmed due to the absence of controlled or comparative data. Future studies employing longitudinal cohort designs or quasi-experimental approaches such as interrupted time series analysis would help establish stronger evidence for the impact of such interventions.

Prophet component analysis in STL and standard decomposition revealed annual peaks in early months (February & March), consistent with earlier researcher [23], who documented peak canine rabies in Chennai from 2011 to 2014 through descriptive analysis. In the earlier study period (from 1973 to 1983), similar peaks were observed in March in the same area, then called Madras [32,33]. This study's detailed decomposition revealed significant peaks in early months followed by variation; as no seasonality was observed, further season or month wise risk analysis was not attempted.

The four prediction models demonstrated varied prediction performance and efficacy with minimized error metrics, indicating each model's methodology impacts data training, fitting and forecasting. The wide confidence intervals observed in models like BSTS highlight forecast uncertainty, particularly in long-range predictions. This uncertainty should be interpreted with caution when using model outputs to inform rabies control strategies. Rather than relying on a single model outcome, a range of projections should be considered to guide flexible and responsive policy decisions. Incorporating such uncertainty into planning can improve preparedness and support more robust, adaptive public health interventions.

We selected RMSE, MAE, MAPE and MASE as evaluation metrics because they provide interpretable and practical measures of forecasting accuracy, especially when comparing models on real-world data [[19], [20], [21]]. While AIC or BIC focus more on model parsimony and fit, our goal was to assess how well each model generalized to unseen data, a key requirement in public health forecasting. For this dataset, the Prophet model performed optimally. The superior performance of the Prophet model in our study may be attributed to its built-in features such as automatic seasonality detection, robust handling of missing data and the ability to model change points in long-term data. These attributes make Prophet particularly effective for datasets with irregular patterns and moderate overdispersion. Our finding is consistent with previous studies that have utilized Prophet successfully for forecasting, including wholesale food prices [34] and hand-foot-mouth disease incidence [35]. While Prophet was optimal for our dataset, variations in model performance are expected depending on dataset structure, as demonstrated in comparative studies of infectious disease forecasting using different time series models such as GAM, BSTS and STL + ARIMA for chronic obstructive pulmonary disease [14], influenza [36,37], COVID-19 [38] and Turkish coal production [39]. These variations reflect different modelling assumptions, offering a range of potential future scenarios for rabies incidence.

Understanding canine rabies epidemiology serves as a critical platform for estimating the burden of human rabies in the region. The insights gained from trends and patterns of canine rabies occurrences highlight the urgent need to explore human rabies data in local settings. This study underscores the interconnectedness between canine rabies surveillance and human public health outcomes, reaffirming the foundational principles of the One Health approach. Since dog-mediated transmission accounts for the vast majority of human rabies cases in Asia, our findings offer a critical baseline for anticipating and preventing human exposures. Integrating canine rabies data with human rabies incidence, dog bite reports and vaccine coverage could help inform more holistic and responsive rabies control policies. Strengthening collaboration between veterinary and public health sectors will be essential for designing and implementing targeted interventions aligned with the national rabies elimination strategies. These insights reinforce the value of cross-sectoral data integration for developing sustainable and regionally tailored disease prevention frameworks.

Establishing the significant association between canine and human rabies is particularly crucial in Asia, where 99 % of human rabies cases are attributed to dog-mediated transmission. Epidemiological studies on canine rabies provide baseline data essential for assessing the true burden of rabies in both humans and animals. Moreover, incorporating human rabies data, dog bite wound cases and associated risk factors will be instrumental in devising effective and targeted control strategies. Achieving the goal of eliminating dog-mediated rabies by 2030 necessitates data-driven approaches that integrate canine and human rabies epidemiology into a unified “One Health” framework. Exploratory studies with a focus on such holistic methodologies will play a vital role in constructing actionable strategies and advancing the global mission to eradicate rabies.

Despite the valuable insights gained from this study in understanding the trends, seasonality and patterns of canine rabies in Chennai, a key limitation is the potential for selection bias due to reliance on hospital-based data. The dataset, derived from laboratory-confirmed cases reported at the Teaching Veterinary Hospital in Madras Veterinary College, Chennai, provides a useful snapshot of canine rabies cases in the region. However, it may not fully reflect the broader population dynamics of rabies in Chennai or other parts in India. This reliance on a single urban referral centre may lead to underrepresentation of cases from rural or underserved areas, where rabies often goes undiagnosed or underreported due to limited access to veterinary care. Moreover, formal sample size estimation was not feasible due to the retrospective nature of the study, but the long-term, complete dataset supports meaningful time series analysis within this context.

Ideally, to better capture the full scope of rabies occurrences and enhance the generalizability of the findings, data collection would need to span a longer time period and incorporate multiple collection points across different geographic regions. Long-term data collection would allow for a more comprehensive understanding of seasonal variations, trends and outbreaks over time, as well as a more accurate representation of the broader epidemiological picture. Additionally, including data from a variety of regions both within India and internationally would improve the ability to extrapolate findings to other areas with similar demographic and environmental characteristics.

Incorporating diverse data sources, such as community-based surveillance or reports from local veterinary clinics, could also help capture a more complete picture of the rabies burden and its dynamics. This would allow for a more robust analysis of the patterns of rabies transmission and provide a stronger foundation for developing targeted and region-specific interventions. Future studies could also consider integrating human rabies case data and zoonotic transmission patterns to further enrich the understanding of rabies epidemiology and enhance the predictive power of the models. While this study offers important insights, addressing these limitations in future research would help create more generalizable conclusions and strengthen the evidence base for effective rabies control strategies.

## 5 Conclusion

This study analyzed laboratory-confirmed canine rabies data from Chennai, India, over a nine-year period (March 2010 to February 2019), revealing a non-seasonal yet declining trend in rabies incidence. Two significant change points were identified, highlighting temporal shifts in case patterns. Among the models evaluated, the Prophet model demonstrated the best predictive performance, supporting its utility for forecasting rabies cases. These findings underscore the importance of long-term surveillance and advanced time series modelling in understanding disease dynamics. To support the goal of eliminating dog-mediated human rabies by 2030, the study recommends strengthening continuous canine rabies surveillance, promoting intersectoral data-sharing between veterinary and public health sectors and enhancing vaccination campaigns, especially in months with higher predicted incidence or greater uncertainty. Implementing such targeted strategies under the One Health framework will be crucial for improving rabies control and prevention outcomes in endemic regions like Chennai, India. Similar studies in other settings are needed to build a global evidence base for rabies elimination efforts.

## Funding

This research did not receive any specific grant from funding agencies in the public, commercial, or not-for-profit sectors.

## CRediT authorship contribution statement

**Viswanathan Naveenkumar:** Writing – review & editing, Writing – original draft, Methodology, Investigation, Formal analysis, Data curation, Conceptualization. **Mangalanathan Vijaya Bharathi:** Writing – review & editing, Supervision, Project administration. **Porteen Kannan:** Writing – review & editing, Writing – original draft, Methodology, Investigation, Formal analysis, Data curation, Conceptualization. **B.S. Pradeep Nag:** Writing – review & editing, Methodology, Formal analysis, Data curation. **Sureshkannan Sundaram:** Writing – review & editing, Methodology, Data curation. **Nithya Quintoil Mohanadasse:** Writing – review & editing, Methodology. **Raghavendra G. Amachawadi:** Writing – review & editing, Methodology. **Muskan Dubey:** Writing – review & editing, Methodology. **Charley A. Cull:** Writing – review & editing. **Chandan Shivamallu:** Writing – review & editing. **Shiva Prasad Kollur:** Writing – review & editing, Resources. **Ravindra P. Veeranna:** Writing – review & editing, Supervision, Resources, Project administration, Funding acquisition, Conceptualization.

## Declaration of competing interest

The authors have declared that no competing interests exist.

## Data Availability

The data used in this study can be obtained from the corresponding author upon reasonable request.

## Appendix A Supplementary data

Supplementary material Image 1
