# Supplementary material for: Epidemiological insights into canine rabies in Chennai: Trends, forecasting and One Health implications
Source: One Health. 2025 Jul 4;21:101128. doi: 10.1016/j.onehlt.2025.101128 (PMC12274299; doi:10.1016/j.onehlt.2025.101128)
Supplement: Supplementary file 1 — Supplementary material [file mmc1.docx]

**Supplementary material**

**2. Materials and Methods**

*2.3. Change point analysis*

To detect change points within the segments of the time series data on rabies occurrences, a binary segmentation technique was applied using the following function [10,11];

$\sum_{i =1}^{m+ 1} [ ∁_{(X_{(ti - 1):ti})} ] + \beta f(m)$ (1)

In this equation, C represents the cost function, calculated using the negative log likelihood for each segment and 𝛽𝑓(𝑚) is a penalty term used to prevent overfitting by limiting the number of detected segments [9].

The process begins by detecting a single change point in the entire dataset. Upon identifying a change point, the data is divided into two subsegments at that point. This procedure is repeated for each subsegment and the analysis continues until no further change points are found.

*2.6. Decomposition of time series data*

The model is represented as follows [10];

$Y_{t}= T_{t} + S_{t} + R_{t}$ (2)

where, *Y_t_* denotes the number of animal rabies cases at time, *T_t_* represents the trend cycle component, *S_t_* is the seasonal component and *R_t_* stands for the remainder or irregular component at the same period.

*2.7. Development of time series models*

2.7.1. Generalized Additive Model (GAM)

The structure of the GAM is as follows [13]:

$g(E({Cases}_{t})) = \alpha+ f (time)$ (3)

where, *g* () is the link function, specifically a log link for the Poisson distribution, which is appropriate for count data, $(E({Cases}_{t}))$ represents the expected number of rabies cases at time *t*, $\alpha$ is the intercept and $f$*(time)* is a smooth function of time, capturing any non-linear trends in rabies cases.

Exponential Family Distribution of this GAM model is detailed below; Response variable (rabies cases) is assumed to follow a Poisson distribution, a suitable choice for count data. In this framework, the probability mass function (PMF) for each count $\mathcal{y}_{i}$​ is given by [14]:

$\mathcal{P(}\mathcal{y}_{i},\theta_{i}, \phi) = \exp\left( \frac{\mathcal{y}_{i} + b (\theta_{i})}{\phi} + c (\mathcal{y}_{i},\phi) \right)$ (4)

where, $\theta_{i}$​ is the natural parameter specific to $\mathcal{y}_{i}$​, $\phi$ is the dispersion parameter, set to 1 for the Poisson distribution, $b ( )$ and $c ( )$ are functions that define the Poisson distribution.

2.7.2. Prophet Model

The Prophet model decomposes the time series into three components: trend $g(t)$, seasonality $s(t)$ and a noise term $\mathcal{E(}t)$​:

$\mathcal{y(}t) = g(t) + s(t\mathcal{) + E(}t)$ (5)

where, $g(t)$ is the trend function, capturing non-linear changes in rabies cases over time, $s(t)$ models seasonality, $\mathcal{E(}t)$ represents the error term, assumed to be normally distributed. The trend component $g(t)$ in Prophet uses a piecewise linear function with automatically selected changepoints, enabling it to adapt to shifts in the time series trend. This approach is beneficial for disease incidence data, where rates may vary over time due to seasonal or external factors.

2.7.3. Bayesian Structural Time Series (BSTS)

The BSTS model is defined by two key equations:

Observation Equation: $\mathcal{y}_{t} = \mu_{t}+\chi_{t}\beta+ S_{t}+\mathcal{e}_{t}, where \mathcal{e}_{t} \sim N (0, \sigma_{\mathcal{e}}^{2})$ (6)

Transition Equation: $\mu_{t+1}= \mu_{t}+\upsilon_{t}, where \upsilon_{t}\sim N (0, \sigma_{\mathcal{e}}^{2})$ (7)

where, $\mathcal{y}_{t}$​: observed rabies cases at time t; $\mu_{t}$​: unobserved local level (trend) term that evolves over time; $\chi_{t}$​: optional covariates (none were included in this study); $\beta$: coefficients for covariates; $S_{t}$​: seasonal component; $\mathcal{e}_{t}$ and $\upsilon_{t}$​: independent Gaussian noise terms.

2.7.4. Seasonal and Trend Decomposition using Loess (STL) combined with an ARIMA model (STL+ARIMA)

$\hat{Y}_{T + h} = \hat{T}_{T + h} + \hat{S}_{T + h} + \hat{R}_{T + h}$ (8)

where, $h$ ≥1 represents the forecast horizon and $T$ is the last observed time point.
